# Coagulation dysfunction in term neonatal sepsis: a prospective cohort study

**DOI:** 10.3389/fped.2026.1859012

**Published:** 2026-08-20

**Authors:** Dang Thi Thu Thuy, Ta Anh Tuan, Nguyen Thi Duyen, Le Thi Ha, Tran Huu Dat, Nguyen Thi Quynh Nga

**Affiliations:** 1 Hanoi Medical University, Hanoi, Vietnam; 2 Vietnam National Children’s Hospital, Hanoi, Vietnam

**Keywords:** neonatal sepsis, nSOFA score, prognosis, rotational thromboelastometry (ROTEM), sepsis-associated coagulopathy, viscoelastic testing

## Abstract

**Objective:**

To characterize clinical features, conventional coagulation profiles, and rotational thromboelastometry (ROTEM) parameters in term neonates with sepsis, and to evaluate their associations with all-cause mortality within 28 days after sepsis diagnosis, with particular emphasis on whether a fibrin-based ROTEM parameter provides incremental prognostic information beyond organ dysfunction severity and conventional coagulation testing.

**Methods:**

This prospective hospital-based cohort study was conducted at the Neonatal Center of Vietnam National Children's Hospital between June 2024 and December 2025. Term neonates (gestational age 37–42 weeks) diagnosed with neonatal sepsis according to the European Medicines Agency 2010 criteria were enrolled. Clinical severity was assessed using the neonatal Sequential Organ Failure Assessment (nSOFA) score. Conventional coagulation tests included platelet count, prothrombin time/international normalized ratio (INR), aPTT ratio, fibrinogen, D-dimer, and selected natural anticoagulants. Viscoelastic coagulation was evaluated using ROTEM (INTEM, EXTEM, and FIBTEM assays). The primary outcome was all-cause mortality within 28 days after sepsis diagnosis. Associations between clinical, laboratory, and ROTEM parameters and mortality were examined using univariable analyses and prespecified multivariable logistic regression models. Model discrimination was assessed using the area under the receiver operating characteristic curve (AUC).

**Results:**

A total of 168 term neonates with sepsis were included, of whom 23 died within 28 days (mortality rate, 13.7%). Non-survivors had significantly higher nSOFA scores at diagnosis than survivors (median 12 vs. 6; *P* < 0.001) and universally presented with septic shock and required mechanical ventilation. Conventional coagulation testing demonstrated a hypocoagulable profile among non-survivors, characterized by lower platelet counts, prolonged PT/INR and aPTT ratio, lower fibrinogen levels, and reduced protein S and antithrombin III activities. ROTEM analyses revealed reduced clot strength (lower A5, A10, and maximum clot firmness) and impaired clot propagation (prolonged clot formation time and reduced alpha angle) across INTEM, EXTEM, and FIBTEM assays. In multivariable logistic regression using complete-case data (*n* = 161; deaths=20), the nSOFA score remained independently associated with mortality (OR per 1-point increase, 1.53; 95% CI, 1.25–1.87), whereas INR was not. Addition of FIBTEM maximum clot firmness (per 10-mm increase) showed a protective direction of association with mortality (OR, 0.43; 95% CI, 0.17–1.09) but resulted in only a modest, non-significant improvement in discrimination (AUC 0.862 vs. 0.878; DeLong *P* = 0.455).

**Conclusion:**

Among term neonates with sepsis, early organ dysfunction severity assessed by nSOFA was the strongest determinant of short-term mortality. Non-survivors exhibited a consistent hypocoagulable phenotype characterized by prolonged conventional coagulation times, reduced clot strength, and impaired clot propagation on ROTEM. Although FIBTEM maximum clot firmness reflected disease severity and showed a clinically meaningful association with mortality, its incremental prognostic value beyond nSOFA and conventional coagulation measures was limited. ROTEM may therefore be most useful as a complementary tool for hemostatic phenotyping and assessment of coagulation dysfunction rather than as a stand-alone mortality prediction instrument. Larger multicenter studies incorporating serial viscoelastic assessments are warranted to further define its prognostic and clinical utility.

## Introduction

Neonatal sepsis remains a major global health problem and a leading cause of neonatal morbidity and mortality, accounting for a substantial proportion of deaths during the first 28 days of life worldwide (1–3). Although advances in antimicrobial therapy and neonatal intensive care have improved survival, mortality among neonates with sepsis remains high, particularly in those who develop severe organ dysfunction (2, 4). Term neonates, often considered at lower risk than preterm infants, may still deteriorate rapidly and die once sepsis progresses, highlighting the importance of early risk stratification in this group (5). This issue is especially relevant in low- and middle-income countries, including Vietnam, where high birth rates, heavy neonatal caseloads, and limited access to advanced intensive care contribute to a substantial burden of severe neonatal sepsis.

Sepsis is characterized by a dysregulated host response to infection that involves complex interactions between inflammation, endothelial injury, and the coagulation system (6, 7). Systemic inflammation may promote endothelial activation, microcirculatory dysfunction, coagulation activation, and impairment of endogenous anticoagulant pathways, ultimately contributing to organ dysfunction and adverse outcomes (6, 7). In neonates, these processes may result in profound disturbances of hemostasis, including activation of coagulation pathways, endothelial dysfunction, and formation of microvascular thrombi, which may subsequently lead to consumption of platelets and coagulation factors and impaired clot formation in severe disease (6, 7). Conventional coagulation tests, such as prothrombin time, activated partial thromboplastin time, and plasma fibrinogen concentration, are widely used in clinical practice but assess isolated components of the coagulation cascade and provide limited information on the dynamic process of clot initiation, propagation, and stability (8). As a result, they may not fully reflect the overall hemostatic changes seen in sepsis-associated coagulopathy, particularly in clinical settings such as Vietnam, where conventional assays remain the primary tools available in most neonatal units and advanced hemostatic testing is not uniformly implemented.

Clinical severity scores have been developed to improve prognostication in sepsis. The neonatal Sequential Organ Failure Assessment (nSOFA) score has been shown to correlate with mortality in neonatal sepsis and provides a practical tool for assessing multiorgan dysfunction (9, 10). However, organ dysfunction scores primarily reflect end-organ injury and may not adequately integrate coagulation abnormalities, which themselves have been associated with adverse outcomes in septic patients (6, 7). Whether a more detailed assessment of coagulation disturbances adds prognostic value beyond established clinical severity scores in term neonates remains unclear, particularly in resource-limited settings where early identification of high-risk patients could help guide escalation of care and use of intensive care resources.

Rotational thromboelastometry (ROTEM) is a viscoelastic whole-blood assay that provides a comprehensive evaluation of coagulation by integrating the contributions of plasma coagulation factors, platelets, and the fibrinolytic system (11). In adult and pediatric critical care, ROTEM has been increasingly applied for the rapid assessment of coagulation disorders and to guide bleeding management and transfusion therapy (12–14). However, evidence regarding ROTEM profiles in neonates, particularly term neonates with sepsis, is limited, and existing studies are heterogeneous in design and outcomes. In addition, the extent to which ROTEM parameters improve prognostic assessment beyond conventional coagulation tests and clinical severity scores has not been clearly established in this population, and data from Vietnam and other Southeast Asian settings remain scarce. Therefore, the objectives of this study were to characterize the clinical characteristics, conventional coagulation profiles, and ROTEM parameters in term neonates with sepsis; to examine the associations between these factors and all-cause mortality within 28 days after sepsis diagnosis; and to evaluate whether the inclusion of ROTEM-derived indices improves the discriminative performance of mortality prediction models beyond clinical severity and conventional coagulation measures.

## Methods

### Study design and setting

This study was conducted at the Neonatal Center, Vietnam National Children's Hospital, a national tertiary referral center in Hanoi, Vietnam. The investigation was embedded within a doctoral research project designed to evaluate coagulation abnormalities and their association with mortality in neonatal sepsis.

The overarching project was planned as a hospital-based observational study, comprising (i) a descriptive component to characterize coagulation disturbances in neonatal sepsis and (ii) a prospective longitudinal component to evaluate prognostic associations with short-term mortality. The present manuscript reports analyses derived from this predefined cohort and adheres to the original study protocol.

### Study population

Eligible participants were term neonates (gestational age 37–42 completed weeks) admitted to the Neonatal Center with a diagnosis of neonatal sepsis between June 2024 and December 2025. Gestational age was determined using obstetric dating records and/or early ultrasound findings documented in the medical record.

Neonatal sepsis was defined according to the European Medicines Agency (EMA) 2010 consensus criteria (15), requiring the presence of at least two clinical signs and at least two laboratory abnormalities consistent with systemic infection. Clinical signs included, but were not limited to, temperature instability, respiratory distress, cardiovascular instability, feeding intolerance, and other non-specific clinical signs (e.g., irritability, lethargy), as well as skin and subcutaneous lesions. Laboratory criteria included abnormalities in inflammatory markers, hematologic indices, or metabolic parameters, as specified in the EMA framework.

Cases were classified as proven infection when blood culture or polymerase chain reaction (PCR) testing from a sterile site yielded a pathogenic organism, and as suspected infection when cultures and/or PCR were negative. Still, the predefined clinical and laboratory criteria for sepsis were fulfilled. Early-onset sepsis was defined as sepsis occurring within the first 72 h of life, whereas late-onset sepsis was defined as sepsis occurring at or beyond 72 h after birth, consistent with contemporary neonatal sepsis definitions (16).

Neonates were excluded if they had major congenital anomalies or chromosomal abnormalities, or if they experienced severe perinatal asphyxia as documented by clinical and biochemical findings in the medical records. Additional exclusion criteria included receipt of plasma products within 3 days or platelet transfusion within 7 days before blood sampling for coagulation assessment; early death within 24 h of sepsis diagnosis precluding standardized coagulation evaluation; or refusal of informed consent by parents or legal guardians.

### Data collection and clinical variables

Data were collected prospectively using standardized case-report forms. Demographic and perinatal variables (including sex, gestational age, birth weight, mode of delivery, maternal risk factors, perinatal complications, and early neonatal interventions) were recorded at admission. Clinical characteristics were assessed at the time of sepsis diagnosis, including presenting symptoms, hemodynamic status, respiratory support, use of vasoactive medications, and major organ dysfunctions. Disease severity was evaluated using the neonatal Sequential Organ Failure Assessment (nSOFA) score, calculated at the time of sepsis diagnosis according to predefined criteria. The nSOFA score evaluates three organ systems: respiratory dysfunction (based on ventilatory support and oxygen requirement), cardiovascular dysfunction (based on vasoactive medication use and corticosteroid therapy), and hematologic dysfunction (based on platelet count), according to the original published definition of the score (9). Septic shock was defined as sepsis accompanied by hypotension requiring fluid resuscitation and/or vasoactive-inotropic support, according to diagnostic criteria used in our NICU and consistent with contemporary neonatal sepsis literature (16).

Laboratory parameters, including hematologic, inflammatory, biochemical, and coagulation variables, were collected during the first 24 h after admission. Because several laboratory investigations were repeated as part of routine clinical care, the values reported in the analyses represent the most abnormal measurement recorded during the first 24-hour period.

### Microbiological evaluation

Microbiological investigations were performed in accordance with routine clinical practice at the Neonatal Center. Blood cultures were obtained for all patients at the time of sepsis evaluation. When clinically indicated, additional cultures and/or PCR testing were performed on specimens from other sterile body fluids, including cerebrospinal fluid, catheterized urine, bronchoalveolar lavage fluid, and peritoneal fluid. Identified microorganisms were recorded and categorized according to standard microbiological classification. For culture-negative cases, additional microbiological specimens were collected when clinically indicated according to the suspected source of infection. However, suspected primary infection sites were not prospectively recorded in a standardized dataset and therefore were not available for formal analysis.

### Conventional coagulation testing

Blood samples for coagulation assessment were obtained within 24 h of sepsis diagnosis and, whenever possible, before administration of blood products. In routine clinical practice, coagulation testing, ROTEM assessment, and blood cultures were generally performed at the time of admission and, whenever feasible, before initiation of antimicrobial therapy. Exact timing relative to blood culture acquisition and first antibiotic administration was not prospectively recorded for all patients. Conventional coagulation tests included platelet count, prothrombin time (PT) expressed as international normalized ratio (INR), activated partial thromboplastin time (aPTT), fibrinogen concentration, D-dimer, and antithrombin activity. These tests were performed as part of routine clinical care in the hospital laboratory, and results were extracted from the electronic medical records.

Whenever feasible, coagulation samples were obtained by peripheral venipuncture or from non-heparinized vascular access. In neonates with existing vascular catheters, blood sampling followed institutional protocols designed to minimize heparin contamination, including discarding an initial blood volume before collection of coagulation specimens.

### Viscoelastic coagulation assessment (ROTEM)

In addition to conventional coagulation testing, viscoelastic hemostatic assessment was performed using rotational thromboelastometry (ROTEM). ROTEM measurements were obtained within 24 h of sepsis diagnosis, contemporaneously with conventional coagulation tests whenever feasible and before transfusion when possible. INTEM, EXTEM, and FIBTEM assays were analyzed to evaluate different coagulation pathways.

ROTEM parameters reflecting clot initiation and propagation [clotting time (CT), clot formation time (CFT)], clot strength [amplitude at 5 and 10 min (A5, A10), maximum clot firmness (MCF)], and fibrinolysis (lysis index) were recorded. Interpretation of ROTEM results was based on age-appropriate neonatal reference ranges derived from published neonatal viscoelastic studies and reference datasets (17, 18). Analyses focused on parameters with established relevance to clot firmness and overall hemostatic competence.

### Outcome

The primary outcome was all-cause mortality within 28 days after the diagnosis of neonatal sepsis. Vital status was determined through in-hospital follow-up and review of medical records.

### Statistical analysis

Continuous variables are reported as medians [interquartile ranges (IQRs)], and categorical variables as counts (percentages). Comparisons between survivors and non-survivors were performed using the Mann–Whitney U test for continuous variables and the *χ*^2^ test or Fisher's exact test for categorical variables, as appropriate. Microbiological results were summarized descriptively for the overall cohort and, where applicable, compared by survival status using *χ*^2^ or Fisher's exact tests. For ROTEM parameters and other laboratory variables, analyses were conducted using an available-case approach. The number of neonates contributing data for each parameter and survival group is reported in the corresponding tables, with “n” for each variable indicating the number of non-missing measurements. This approach was chosen because missingness primarily reflected logistical or operational constraints in obtaining ROTEM testing rather than protocol-defined sampling.

Multivariable logistic regression was performed using a complete-case approach for all prespecified predictors; consequently, neonates with missing values for any model covariate were excluded from these analyses. Of the 168 neonates included in the study (23 deaths), 161 (95.8%) had complete data available for all variables included in the multivariable models and were therefore retained for regression analyses. Seven neonates were excluded because of missing data in at least one model covariate, resulting in the exclusion of three deaths and four survivors. The final complete-case dataset thus comprised 161 neonates, including 20 deaths, and was used for all multivariable analyses presented in Table 7. Because the proportion of missing data was small, no imputation procedures were performed. To evaluate factors independently associated with all-cause mortality within 28 days after sepsis diagnosis and to assess the incremental prognostic value of ROTEM beyond clinical severity and conventional coagulation parameters, a hypothesis-driven, stepwise modeling strategy was applied, resulting in two prespecified models. Model 1 was defined as the baseline model and included the neonatal Sequential Organ Failure Assessment (nSOFA) score and international normalized ratio (INR), representing clinical severity and conventional coagulation status. These variables were selected *a priori* based on clinical relevance and supported by findings from earlier analyses (Tables 1–3). Model 2 extended Model 1 by incorporating a ROTEM-derived parameter, FIBTEM maximum clot firmness (MCF), to evaluate whether viscoelastic testing provides additional prognostic information. FIBTEM MCF was selected based on its biological relevance to fibrin-based clot strength and its strong association with mortality observed in Table 4. To facilitate clinical interpretability, FIBTEM MCF was scaled per 10-mm increase. This two-model framework enabled direct comparison between a clinically based model and a model incorporating ROTEM, thereby quantifying the incremental prognostic value of viscoelastic testing. Because the number of deaths was limited, the primary multivariable analysis was restricted to these prespecified predictors to reduce the risk of overfitting.

**Table 1:** Baseline demographic and clinical characteristics of term neonates with sepsis according to survival status.

Variable	Overall (*n* = 168)	Survivors (*n* = 145)	Non-survivors (*n* = 23)	*p-*value
Gestational age, weeks	38.0 (38.0–39.0)	38.0 (38.0–39.0)	39.0 (37.0–40.0)	0.438
Birth weight, g	3,100 (2,900–3,500)	3,100 (2,900–3,500)	3,100 (3,000–3,500)	0.831
Length of hospital stay until discharge or death, days	13.0 (10.0–21.0)	14.0 (11.0–22.0)	3.0 (2.0–10.0)	<0.001
nSOFA score	6.0 (2.0–10.0)	6.0 (0.0–8.0)	12.0 (8.0–12.0)	<0.001
Mode of delivery				0.030
Vaginal delivery	61 (36.3)	48 (33.1)	13 (56.5)	
Cesarean section	107 (63.7)	97 (66.9)	10 (43.5)	
Sepsis onset				0.304
Early-onset sepsis	116 (69.0)	98 (67.6)	18 (78.3)	
Late-onset sepsis	52 (31.0)	47 (32.4)	5 (21.7)	
Septic shock				<0.001
No	78 (46.4)	78 (53.8)	0 (0.0)	
Yes	90 (53.6)	67 (46.2)	23 (100.0)	
Mechanical ventilation				0.001
No	43 (25.6)	43 (29.7)	0 (0.0)	
Yes	125 (74.4)	102 (70.3)	23 (100.0)	
Bleeding events				0.090
No	134 (79.8)	119 (82.1)	15 (65.2)	
Yes	34 (20.2)	26 (17.9)	8 (34.8)	
Thrombotic events				0.055
No	161 (95.8)	141 (97.2)	20 (87.0)	
Yes	7 (4.2)	4 (2.8)	3 (13.0)	

Data are presented as median (interquartile range) or number (percentage). Comparisons between survivors and non-survivors were performed using the Mann–Whitney U test for continuous variables and the chi-square test or Fisher's exact test, as appropriate. nSOFA, neonatal Sequential Organ Failure Assessment score. Early-onset sepsis was defined as sepsis occurring within the first 72 h of life, and late-onset sepsis as sepsis occurring thereafter. A two-sided *p* value < 0.05 was considered statistically significant.

**Table 2:** Hematological, inflammatory, and biochemical parameters of term neonates with sepsis according to survival status.

Variable	Overall (*n* = 168)	Survivors (*n* = 145)	Non-survivors (*n* = 23)	*p-*value
White blood cell count, ×10⁹/L	15.06 (9.37–21.58)	15.00 (9.59–21.00)	16.27 (7.06–25.93)	0.897
Absolute neutrophil count, ×10⁹/L	9.49 (5.78–15.23)	9.50 (5.91–15.19)	9.48 (4.45–19.71)	0.969
Platelet count, ×10⁹/L	237 (161–301)	239 (166–309)	205 (112–265)	0.029
Hemoglobin, g/L	142.5 (125.5–159.5)	142.0 (125.0–158.0)	148.0 (128.0–170.0)	0.277
C-reactive protein, mg/L	30.68 (6.74–86.34)	31.56 (7.06–88.00)	23.90 (2.67–52.00)	0.290
Serum albumin, g/L	30.80 (27.60–32.90)	30.80 (27.90–32.70)	30.75 (23.20–33.80)	0.423
Total protein, g/L	48.45 (43.60–54.00)	48.40 (44.20–54.00)	49.45 (34.20–54.80)	0.426
Aspartate aminotransferase (AST), U/L	71.30 (41.90–112.70)	64.90 (37.70–106.00)	111.10 (71.20–247.00)	<0.001
Alanine aminotransferase (ALT), U/L	13.10 (9.00–20.45)	13.10 (9.00–20.20)	13.20 (9.10–24.60)	0.512
Blood urea, mmol/L	4.70 (3.40–6.80)	4.60 (3.25–6.75)	5.00 (4.30–6.80)	0.303
Serum creatinine, µmol/L	64.80 (42.90–85.50)	61.75 (38.50–82.55)	78.80 (67.20–88.20)	0.007

Data are presented as median (interquartile range). Comparisons between survivors and non-survivors were performed using the Mann–Whitney U test. WBC, white blood cell count; AST, aspartate aminotransferase; ALT, alanine aminotransferase. A two-sided *p* value <0.05 was considered statistically significant.

**Table 3:** Conventional coagulation parameters and endogenous anticoagulants in term neonates with sepsis according to survival status.

Variable	Overall (*n* = 168)	Survivors (*n* = 145)	Non-survivors (*n* = 23)	*p-*value
Prothrombin activity, %	62 (48–79)	64 (52–81)	42.5 (35–65)	0.002
Prothrombin time, s	16.1 (13.9–19.9)	15.85 (13.6–18.7)	22.9 (17–25.8)	<0.001
INR	1.40 (1.17–1.73)	1.36 (1.15–1.63)	1.91 (1.32–2.21)	0.002
aPTT ratio	1.37 (1.16–1.61)	1.36 (1.15–1.54)	1.72 (1.35–2.15)	0.001
Fibrinogen, g/L	3.01 (2.12–4.28)	3.18 (2.27–4.36)	2.08 (1.63–2.98)	0.002
D-dimer, ng/mL	2,670 (1,502–6,470)	2,524 (1,500–5,880)	4,531 (1,770–10,244)	0.093
Protein C activity, %	21 (13–29)	21.5 (13.75–29.5)	15 (11–24)	0.153
Protein S activity, %	37.05 (26.35–54.3)	39.2 (29.1–56.4)	23.5 (15.1–32.8)	<0.001
Antithrombin III activity, %	41 (31–52)	42 (31.5–53.5)	34 (24–44)	0.032

Data are presented as median (interquartile range). Comparisons between survivors and non-survivors were performed using the Mann–Whitney U test. INR, international normalized ratio; aPTT, activated partial thromboplastin time. A two-sided *p* value <0.05 was considered statistically significant.

**Table 4:** Rotational thromboelastometry (ROTEM) parameters in term neonates with sepsis according to survival status.

Variable	Overall	Survivors	Non-survivors	*p-*value
INTEM				
Clotting time (CT), s	219.5 (186–276)	220.0 (186–272)	208.0 (180–342)	0.676
Clot formation time (CFT), s	115.0 (80–148)	108.0 (80–144)	144.5 (120–224)	0.007
Alpha angle (*α*),°	69.0 (63–74)	70.0 (63–74)	58.5 (35–68)	<0.001
A5, mm	37.0 (30–45)	38.0 (33–45)	29.0 (13–33)	<0.001
A10, mm	47.0 (41–56)	49.0 (43–57)	40.0 (21–44)	<0.001
MCF, mm	55.0 (50–61)	56.0 (51–61)	48.0 (28–55)	0.004
ML, %	10.0 (8–13)	10.0 (8–14)	4.5 (2–11)	<0.001
LI30, %	100 (99–100)	100 (99–100)	100 (100–100)	0.041
LI60, %	94.0 (91–97)	94.0 (91–96)	98.5 (95–100)	<0.001
TPI	32.0 (20–57)	33.0 (22–57)	21.5 (13–35)	0.018
EXTEM				
Clotting time (CT), s	61.0 (52.5–74.5)	59.0 (50–70)	76.0 (61–121)	<0.001
Clot formation time (CFT), s	120.5 (87–168)	115.0 (84–161)	188.0 (120–264)	0.008
Alpha angle (α),°	67.5 (60–74)	69.0 (62–75)	53.0 (38–70)	<0.001
A5, mm	36.0 (29–46)	37.0 (31–46)	25.5 (11–32)	<0.001
A10, mm	47.0 (40–56)	48.0 (41–56)	36.5 (17–44)	<0.001
MCF, mm	55.0 (50–62)	56.0 (51–62)	46.5 (24–57)	0.001
ML, %	12.0 (8–16)	12.0 (8–16)	6.0 (1–11)	0.002
LI30, %	100 (99–100)	100 (99–100)	100 (100–100)	0.161
LI60, %	94.0 (90–97)	94.0 (90–97)	97.5 (94–100)	<0.001
TPI	32.0 (18–56)	33.0 (20–56)	15.0 (8–36)	0.015
FIBTEM				
Clotting time (CT), s	57.0 (49–71)	56.0 (48–69)	80.0 (59–103)	<0.001
A5, mm	12.0 (8–18)	13.0 (9–19)	6.5 (5–11)	<0.001
A10, mm	14.0 (9–20)	14.0 (10–22)	7.5 (5.5–12)	<0.001
MCF, mm	16.0 (11–23)	16.5 (12–24)	9.0 (6–16.5)	0.001
ML, %	0.0 (0–3)	0.0 (0–3)	0.0 (0–1)	0.555
LI30, %	100 (100–100)	100 (100–100)	100 (100–100)	0.443
LI60, %	100 (98–100)	100 (98–100)	100 (100–100)	0.176

Data are presented as median (interquartile range). Comparisons between survivors and non-survivors were performed using the Mann–Whitney U test. INTEM, intrinsic pathway activation; EXTEM, extrinsic pathway activation; FIBTEM, fibrin-based clot assessment; CT, clotting time; CFT, clot formation time; A5/A10, clot amplitude at 5 and 10 min; α angle, clot propagation angle; MCF, maximum clot firmness; ML, maximum lysis; LI30/LI60, lysis index at 30 and 60 min; TPI, thrombin potential index. A two-sided *P* value <0.05 was considered statistically significant.

Model discrimination was assessed using the area under the receiver operating characteristic curve (AUC). AUC values were calculated for each model, and differences between correlated ROC curves were formally compared using DeLong's test to evaluate whether the addition of ROTEM parameters significantly improved predictive performance. Sensitivity analyses using penalized logistic regression were not performed; however, model robustness was supported by consistent effect estimates across models and the limited number of predictors relative to the number of outcome events.

All statistical tests were two-sided, and a *p* value <0.05 was considered statistically significant. Analyses were performed using Stata, version 16.0 (StataCorp).

## Results

Table 1 summarizes the baseline demographic and clinical characteristics of the 168 term neonates with sepsis included in this prospective cohort. Overall mortality was 13.7% (23/168). Gestational age and birth weight were comparable between survivors and non-survivors (median gestational age: 38 vs. 39 weeks, *p* = 0.438; median birth weight: 3,100 g in both groups, *p* = 0.831). Mode of delivery differed between groups, with vaginal delivery being more frequent among non-survivors than survivors (56.5% vs. 33.1%, *p* = 0.030). In contrast, disease severity differed markedly. Non-survivors had significantly higher nSOFA scores than survivors [median 12 (IQR 8.0–12.0) vs. 6 (0.0–8.0), *p* < 0.001]. All non-survivors presented with septic shock (100% vs. 46.2% in survivors, *p* < 0.001) and required mechanical ventilation (100% vs. 70.3%, *p* = 0.001). The length of hospital stay until death or discharge was significantly shorter in non-survivors than in survivors [median 3 (2.0–10.0) vs. 14 (11.0–22.0) days; *p* < 0.001], reflecting earlier mortality among non-survivors. The distribution of early- and late-onset sepsis did not differ significantly between groups. Similarly, bleeding and thrombotic events were numerically more frequent among non-survivors, although these differences did not reach statistical significance (*p* = 0.090 and *p* = 0.055, respectively).

Hematological, inflammatory, and biochemical parameters are presented in Table 2. White blood cell count, absolute neutrophil count, hemoglobin concentration, C-reactive protein, albumin, and total protein levels were similar between survivors and non-survivors. However, platelet counts were significantly lower in non-survivors [median 205 × 10⁹/L (112–265) vs. 239 × 10⁹/L (166–309), *p* = 0.029]. Markers of organ dysfunction showed significant differences: non-survivors had markedly higher aspartate aminotransferase levels [111.1 (71.2–247.0) vs. 64.9 (37.7–106.0) U/L, *p* < 0.001] and higher serum creatinine concentrations [78.8 (67.2–88.2) vs. 61.75 (38.5–82.55) µmol/L, *p* = 0.007]. Other biochemical parameters did not differ significantly between groups.

Table 3 compares conventional coagulation parameters between survivors and non-survivors. Non-survivors demonstrated significantly impaired coagulation profiles, including lower prothrombin activity [42.5% (35–65) vs. 64.0% (52.0–81.0), *p* = 0.002], prolonged prothrombin time [22.9 (17–25.8) vs. 15.85 (13.6–18.7) seconds, *p* < 0.001], and higher INR values [1.91 (1.32–2.21) vs. 1.36 (1.15–1.63), *p* = 0.002]. The aPTT ratio was significantly prolonged in non-survivors [1.72 (1.35–2.15) vs. 1.36 (1.15–1.54), *p* = 0.001]. Plasma fibrinogen levels were lower in non-survivors [2.08 (1.63–2.98) vs. 3.18 (2.27–4.36) g/L, *p* = 0.002]. Among endogenous anticoagulants, protein S and antithrombin III levels were significantly reduced in non-survivors, while protein C levels showed no significant difference. D-dimer concentrations tended to be higher in non-survivors but did not reach statistical significance.

ROTEM parameters assessed by INTEM, EXTEM, and FIBTEM are presented in Table 4. In INTEM analysis, non-survivors exhibited a significantly prolonged clot formation time [median 144.5 (120–224) vs. 108.0 (80–144) seconds, *p* = 0.007], along with markedly reduced clot amplitude at 5 min [A5: 29.0 (13–33) vs. 38.0 (33–45)mm, *p* < 0.001], at 10 min [A10: 40.0 (21–44) vs. 49.0 (43–57) mm, *p* < 0.001], a significantly lower alpha angle [58.5 (35–68) vs. 70.0 (63–74) degrees, *p* < 0.001], and lower maximum clot firmness [MCF: 48.0 (28–55) vs. 56.0 (51–61) mm, *p* = 0.004]. Similar patterns were observed in EXTEM, where non-survivors demonstrated a significantly lower alpha angle and significantly reduced clot amplitude (A5, A10) and MCF, indicating impaired clot propagation and reduced clot strength. Additionally, EXTEM clotting time and clot formation time were significantly prolonged in non-survivors [CT: 76.0 (61–121) vs. 59.0 (50–70) s, *p* < 0.001; CFT: 188.0 (120–264) vs. 115.0 (84–161) s, *p* = 0.008]. In FIBTEM, clot amplitude and clot firmness were also significantly lower in non-survivors, suggesting reduced fibrin-based clot formation. FIBTEM clotting time was also significantly prolonged in non-survivors [80.0 (59–103) vs. 56.0 (48–69) s, *p* < 0.001]. Regarding fibrinolysis, lysis parameters showed statistically significant but quantitatively small differences between groups. In INTEM and EXTEM, non-survivors had higher lysis indices at 30 and 60 min (LI30 and LI60) and lower maximum lysis (ML), while overall lysis activity remained limited in both groups, with values clustered near the upper range of lysis indices. No significant differences in lysis parameters were observed in FIBTEM.

 Table 5 shows the microbiological results according to survival status among term neonates with sepsis. Overall, microbiological results were available for all 168 neonates. Most cases were culture-negative (127 of 168, 75.6%), while 41 neonates (24.4%) had culture-confirmed infection. The proportion of culture-positive sepsis did not differ significantly between survivors and non-survivors (24.1% vs. 26.1%, respectively; *P* = 0.908). Among the 41 culture-positive cases, Gram-negative bacteria predominated, accounting for 22 cases (53.7%), whereas Gram-positive bacteria were identified in 19 cases (46.3%). No significant differences in the distribution of culture positivity were observed according to survival status.

**Table 5:** Microbiological results according to survival status in term neonates with sepsis.

Microbiological results	Overall (*n* = 168)	Survivors (*n* = 145)	Non-survivors (*n* = 23)	*p-*value
Negative	127 (75.6)	110 (75.9)	17 (73.9)	0.908
Positive	41 (24.4)	35 (24.1)	6 (26.1)	

Data are presented as number (percentage). Comparisons were performed using the *χ*^2^ test or Fisher exact test, as appropriate.

 Table 6 shows the distribution of culture sites and causative pathogens among the 41 culture-positive term neonates with sepsis. Blood was the most common culture site, accounting for 20 cases (48.8%). Other sources included respiratory tract specimens obtained via endotracheal aspirate in 7 cases (17.1%) and bronchoalveolar lavage fluid in 1 case (2.4%), purulent specimens from abscesses in 5 cases (12.2%), cerebrospinal fluid in 5 cases (12.2%), peritoneal fluid in 3 cases (7.3%), and pleural fluid in 1 case (2.4%). Overall, Gram-negative bacteria accounted for 22 of the 41 culture-positive isolates (53.7%), whereas Gram-positive bacteria accounted for 19 isolates (46.3%). Regarding identified pathogens, *Staphylococcus aureus* was the most frequently identified organism, accounting for 11 cases (26.8%). Group *B Streptococcus* was detected in 6 cases (14.6%). Among Gram-negative pathogens, *Escherichia coli* and *Klebsiella pneumoniae* were each identified in 4 cases (9.8%), while *Enterobacter cloacae* complex and *Elizabethkingia meningoseptica* were detected in 3 cases each (7.3%). Less frequently identified organisms included *Enterococcus faecalis, Klebsiella oxytoca*, and *Staphylococcus epidermidis*, each accounting for 1 case (2.4%). Other organisms, grouped for presentation, accounted for 7 cases (17.1%).

**Table 6:** Distribution of causative pathogens in culture-positive term neonates with sepsis (*N* = 41).

Culture sites	n (%)
Blood	20 (48.8)
Respiratory tract – Endotracheal aspirate	7 (17.1)
Respiratory tract – Bronchoalveolar lavage fluid	1 (2.4)
Pleural fluid	1 (2.4)
Cerebrospinal fluid	5 (12.2)
Peritoneal fluid	3 (7.3)
Purulent specimens (abscess)	5 (12.2)
Gram classification	
Gram-negative bacteria	22 (53.7)
Gram-positive bacteria	19 (46.3)
Identified pathogens	
*Staphylococcus aureus*	11 (26.8)
*Staphylococcus epidermidis*	1 (2.4)
*Group B Streptococcus*	6 (14.6)
*Enterococcus faecalis*	1 (2.4)
*Escherichia coli*	4 (9.8)
*Klebsiella pneumoniae*	4 (9.8)
*Klebsiella oxytoca*	1 (2.4)
*Enterobacter cloacae complex*	3 (7.3)
*Elizabethkingia meningoseptica*	3 (7.3)
Other organisms (grouped)	7 (17.1)

Data are presented as number (percentage); Percentages were calculated using the number of culture-positive cases (*N* = 41) as the denominator; Organism names were standardized for presentation based on microbiology records.

**Figure 1:**
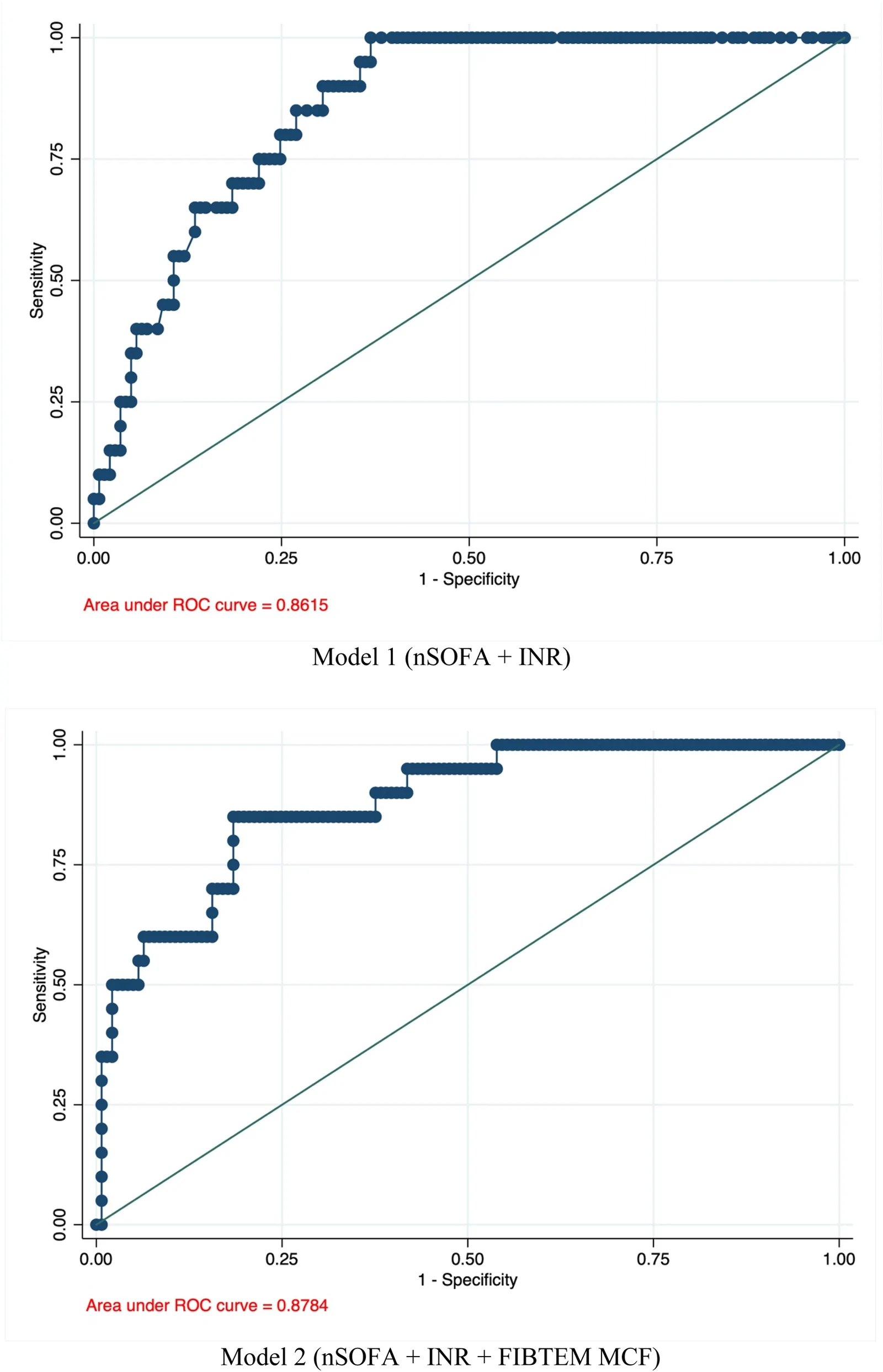
Receiver operating characteristic (ROC) curves for prediction of all-cause mortality within 28 days after sepsis diagnosis using Model 1 (nSOFA + INR) and Model 2 (nSOFA + INR + FIBTEM MCF).

The results of multivariable logistic regression analyses for all-cause mortality within 28 days after sepsis diagnosis are presented in Table 7. Analyses were performed using a complete-case dataset comprising 161 term neonates with sepsis, including 20 deaths. In Model 1, which included disease severity and conventional coagulation parameters, the nSOFA score was independently associated with all-cause mortality within 28 days after sepsis diagnosis. Each 1-point increase in nSOFA was associated with a 53% increase in the odds of death (OR 1.53; 95% CI 1.25–1.87; *p* < 0.001). In contrast, INR was not significantly associated with mortality after adjustment for disease severity (OR 1.08; 95% CI 0.80–1.44; *p* = 0.622). In Model 2, after additional adjustment for the ROTEM-derived parameter FIBTEM maximum clot firmness (MCF), the association between nSOFA and mortality remained robust (OR 1.50; 95% CI 1.22–1.86; *p* < 0.001), while INR remained non-significant (OR 1.03; 95% CI 0.74–1.43; *p* = 0.879). Higher FIBTEM MCF, scaled per 10-mm increase, was associated with lower odds of mortality (OR 0.43; 95% CI 0.17–1.09), although this association did not reach statistical significance (*p* = 0.075). Overall model fit improved modestly after inclusion of FIBTEM MCF, with the pseudo R^2^ increasing from 0.2696 in Model 1 to 0.3004 in Model 2. Model discrimination analyses showed that Model 1 demonstrated good predictive performance, with an area under the receiver operating characteristic curve (AUC) of 0.862. After addition of FIBTEM MCF, Model 2 showed a slightly improved AUC of 0.878, corresponding to a small absolute increase of 0.016 (Figure 1). Formal comparison of the two correlated ROC curves using DeLong's test showed no statistically significant difference between the models (*χ*^2^ = 0.56, *p* = 0.455), indicating that the observed improvement in AUC was not statistically significant. This improvement in discrimination was modest, indicating that incorporation of the ROTEM-derived parameter provided limited incremental predictive value beyond clinical severity and conventional coagulation parameters.

**Table 7:** Multivariable logistic regression models for all-cause mortality within 28 days after sepsis diagnosis.

Variable	Model 1	*p-*value	Model 2	*p-*value
	OR (95% CI)		OR (95% CI)	
nSOFA (per 1-point increase)	1.53 (1.25–1.87)	<0.001	1.50 (1.22–1.86)	<0.001
INR (per 1-unit increase)	1.08 (0.80–1.44)	0.622	1.03 (0.74–1.43)	0.879
FIBTEM MCF (per 10-mm increase)	—	—	0.43 (0.17–1.09)	0.075
Constant	0.003 (0.0003–0.0290)	<0.001	0.013 (0.0009–0.1824)	0.001

Population: Complete-case analysis (*n* = 161; deaths = 20). Model 1 included nSOFA and INR. Model 2 included Model 1 plus FIBTEM maximum clot firmness (MCF), scaled per 10-mm increase. Odds ratios were estimated using multivariable logistic regression. In both models, higher nSOFA was independently associated with higher odds of all-cause mortality within 28 days after sepsis diagnosis. INR was not independently associated with mortality. In Model 2, higher FIBTEM MCF showed an inverse association with mortality, but this did not reach conventional statistical significance.

## Discussion

In this prospective hospital-based cohort of 168 term neonates with sepsis, the 28-day all-cause mortality rate was 13.7%. Three findings are most informative. First, early disease severity at the time of diagnosis, quantified by the neonatal Sequential Organ Failure Assessment (nSOFA) score, showed strong separation between survivors and non-survivors and remained the dominant predictor in parsimonious multivariable models. This is consistent with prior work showing that nSOFA is associated with infection-related mortality in neonates, particularly in preterm infants with late-onset infection, and with pediatric sepsis guidance emphasizing organ dysfunction as the key determinant of sepsis severity and an important anchor for early management and risk stratification (19, 20). Second, non-survivors exhibited a consistent pattern of sepsis-associated coagulation dysfunction across conventional coagulation tests and viscoelastic assays, with a predominant phenotype of impaired clot propagation and reduced clot strength rather than a hyperfibrinolytic pattern. Third, a fibrin-based ROTEM parameter (FIBTEM MCF) showed a clinically meaningful direction of association with mortality; however, its incremental prognostic value beyond clinical severity (nSOFA) and conventional coagulation (INR) was modest when evaluated using AUC-based discrimination, likely reflecting both the dominant signal of organ dysfunction and limited event numbers.

The marked difference in nSOFA scores between survivors and non-survivors underscores that, in term neonates, mortality is primarily coupled to the extent of early organ dysfunction. This finding is consistent with the conceptual framework of pediatric sepsis, in which sepsis is defined by infection-associated life-threatening organ dysfunction, and with the multicenter study by Fleiss et al., where higher nSOFA scores were associated with mortality across several early assessment time points (19, 20). However, the study by Fleiss et al. was conducted in preterm very-low-birth-weight infants with late-onset infection; therefore, our findings extend this prognostic signal into a distinct population of term neonates with clinically diagnosed sepsis rather than directly replicating that cohort (19). In this cohort, the combination of higher nSOFA, universal septic shock, and universal mechanical ventilation among non-survivors suggests that once systemic derangements are established, deterioration can be rapid even in term infants. The shorter length of hospital stay among non-survivors primarily reflects earlier death following sepsis diagnosis rather than differences in treatment duration. The concordance between mortality and biochemical indicators of organ dysfunction (including AST and creatinine) further supports that early multiorgan involvement captures risk beyond what can be explained by individual laboratory biomarkers. From a mechanistic perspective, this pattern aligns with current concepts of sepsis as a dysregulated host response to infection, characterized by concurrent pro- and anti-inflammatory activation, endothelial dysfunction, microcirculatory derangement, and downstream metabolic and mitochondrial injury that collectively contribute to progressive organ failure (21). Our findings should also be interpreted in the context of previous neonatal prognostic models. Although nSOFA emerged as the dominant predictor in our cohort, it is not the only neonatal severity assessment tool that has been evaluated in critically ill neonates. Previous studies comparing modified NEOMOD, SNAP II, SNAPPE, and SNAPPE II demonstrated that thromboelastometry-derived parameters, particularly EXTEM A10, may provide additional prognostic information beyond clinical severity scores alone (22). Similarly, the NeoSeD score incorporated EXTEM A10 among its strongest predictors of neonatal sepsis and septic shock, supporting the biological relevance of viscoelastic assessment in neonatal risk stratification (23).

Non-survivors demonstrated prolonged PT/INR and prolonged aPTT ratio, lower fibrinogen concentrations, thrombocytopenia, and reduced activity of endogenous anticoagulants, particularly protein S and antithrombin III. This pattern is compatible with sepsis-induced coagulopathy, in which systemic activation of coagulation together with fibrinolytic suppression contributes to organ dysfunction in the setting of systemic inflammation (21, 24). In this framework, endothelial injury is not merely an accompanying feature: proinflammatory stimulation shifts the endothelium toward a procoagulant phenotype through decreased thrombomodulin and heparan sulfate expression and increased tissue factor expression, thereby promoting thrombin generation and microvascular thrombosis (21). In parallel, depletion of physiologic anticoagulant pathways is biologically plausible in severe sepsis, as reductions in antithrombin, protein C, and tissue factor pathway inhibitor have been described during progression of sepsis-induced coagulopathy and DIC (24). In neonates, however, interpretation of reduced anticoagulant activity requires caution because developmental hemostasis is characterized by age-related differences in multiple coagulation proteins, and adult reference thresholds cannot be directly applied (25). Recent neonatal studies have further highlighted the clinical importance of sepsis-associated coagulopathy. In a large NICU cohort, abnormal coagulation parameters were identified in approximately 80% of evaluated sepsis episodes and were associated with greater illness severity, higher nSOFA scores, increased use of vasoactive support, and higher mortality (26). Similarly, thrombocytopenia and coagulation abnormalities have consistently emerged as major predictors of adverse outcomes in neonatal sepsis cohorts (27). Together, these studies support the concept that coagulation abnormalities represent an integral component of disease severity rather than isolated laboratory abnormalities.

Assessment of coagulation in neonates requires explicit consideration of developmental hemostasis, because the levels of many coagulation proteins vary predictably with age and neonatal values cannot be interpreted using adult reference thresholds (25). Within this term-only cohort, however, the survivor–non-survivor differences remain clinically informative: lower fibrinogen and platelet counts, together with prolonged PT/INR and prolonged aPTT ratio, and reduced antithrombin and protein S activity, suggest more severe coagulation dysfunction in fatal cases. This pattern is biologically consistent with sepsis-associated coagulopathy, in which systemic inflammation activates coagulation, suppresses physiologic anticoagulant pathways, and promotes endothelial dysfunction and microvascular thrombosis (21, 28). In this context, the reductions in antithrombin and protein S observed in non-survivors are more likely to reflect impairment of endogenous anticoagulant pathways during severe sepsis rather than differences based on adult-derived abnormality thresholds (25, 28).

The absence of a strong mortality association with D-dimer in this cohort may reflect limited statistical power, the use of a single early sampling window within 24 h after diagnosis, and between-patient heterogeneity in fibrin turnover. This finding is biologically plausible because sepsis-associated coagulopathy is often characterized by impaired or suppressed fibrinolysis rather than overt fibrinolytic activation, which may reduce the discriminatory value of fibrin degradation markers at an early disease stage (29).

Viscoelastic testing provided a whole-blood functional assessment that complemented plasma-based assays. In our cohort, non-survivors demonstrated prolonged clot formation time together with lower A5, A10, alpha angle, and maximum clot firmness across INTEM and EXTEM, indicating slower clot propagation and weaker clot strength. A similar hypocoagulable viscoelastic pattern has been described in septic patients with greater illness severity, where clot formation time is prolonged and alpha angle and maximum clot firmness are reduced, with these parameters correlating with organ dysfunction (30). Importantly, interpretation of neonatal viscoelastic results should be based on age-appropriate reference ranges, as healthy term neonates exhibit distinct developmental hemostasis and ROTEM profiles that differ from those of older children and adults (17). Our results are broadly concordant with previous neonatal thromboelastometry studies. Sokou et al. demonstrated that septic neonates exhibited a hypocoagulable ROTEM profile characterized by reduced clot amplitude and impaired clot firmness compared with healthy controls (31). Similarly, Lampridou et al. reported that viscoelastic abnormalities in neonatal sepsis were associated with impaired clot formation and reduced clot strength, supporting the concept that progressive hypocoagulability accompanies increasing disease severity (32). Taken together, these findings are consistent with our observations, in which non-survivors exhibited delayed clot propagation and reduced clot firmness across both INTEM and EXTEM assays.

Our findings should be interpreted alongside previous neonatal studies suggesting that thromboelastometry-derived parameters may contribute to sepsis assessment. The NeoSeD score incorporated EXTEM A10 among its strongest predictors, supporting the biological and clinical relevance of clot amplitude in neonatal sepsis stratification (23). Similarly, comparative studies of neonatal severity scores have suggested that ROTEM parameters, particularly EXTEM A10, may enhance prognostic performance in models that do not otherwise incorporate biomarkers of coagulopathy (22). The present study extends this literature in several respects. First, our cohort was restricted to term neonates. Second, our primary outcome was all-cause mortality within 28 days after sepsis diagnosis rather than diagnosis of sepsis or septic shock. Third, our incremental prognostic analysis specifically evaluated whether a fibrin-based parameter adds prognostic information beyond nSOFA and INR. Based on its biological relevance and its significant association with mortality in our cohort, FIBTEM MCF was selected for evaluation in the incremental prognostic model. This choice was biologically motivated because FIBTEM more directly reflects fibrin-based clot strength, which was markedly reduced among non-survivors. Our findings are also broadly consistent with the mortality study by Sokou et al. (33), who reported that septic neonates who died during hospitalization exhibited a significantly more hypocoagulable ROTEM profile and identified INTEM MCF as the best-performing ROTEM mortality predictor. Although INTEM MCF rather than FIBTEM MCF emerged as the strongest parameter in their cohort, both studies support the concept that impaired clot firmness reflects greater disease severity and worse outcomes in neonatal sepsis.

In contrast, fibrinolysis-related ROTEM parameters showed only modest differences between survivors and non-survivors, with values remaining within the expected reference range. Therefore, our findings do not suggest overt hyperfibrinolysis as a dominant feature in this cohort. This finding is concordant with current concepts of sepsis-associated coagulopathy, in which fibrinolysis is frequently suppressed while coagulation activation persists, thereby promoting microvascular thrombosis and organ dysfunction (21, 34, 35). Notably, impaired fibrinolysis has been linked to increased mortality in septic shock, supporting the biological plausibility of our observations (34). Together, these findings suggest that fatal neonatal sepsis is characterized by a functionally hypocoagulable whole-blood profile superimposed on a biologically prothrombotic state, where coagulation activation, reduced fibrinolysis, and consumption of platelets and coagulation factors may coexist (21, 35). Nevertheless, neonatal ROTEM phenotypes appear heterogeneous across studies. Differences in gestational age, pathogen distribution, timing of ROTEM assessment, illness severity, transfusion exposure, and outcome definitions may contribute to variability in reported findings. For example, studies of preterm neonates with Gram-positive sepsis have described features of sepsis-associated coagulopathy that differ from the predominantly hypocoagulable pattern observed in critically ill term neonates, while fungal sepsis has also been associated with distinctive thromboelastometric abnormalities. Therefore, individual ROTEM parameters should be interpreted within the broader clinical and biological context rather than viewed as universal markers across all neonatal sepsis populations.

In multivariable analyses, nSOFA remained independently associated with mortality, while INR did not retain significance after adjustment. The lack of an independent association for INR is plausible because INR may partly reflect downstream consequences of severe systemic illness, including shock-related hepatic dysfunction, that overlap with the organ dysfunction already captured by nSOFA. Adding FIBTEM MCF resulted in only a small, non-significant increase in AUC. In this study, the absolute increase in AUC was small and statistically non-significant, reinforcing that ROTEM added limited incremental predictive value beyond nSOFA. Several considerations may explain this finding. First, when the baseline model already contains a strong predictor such as nSOFA, the incremental gain from adding a single hemostatic parameter may be limited. Second, our complete-case multivariable analysis included only 20 deaths, and small event numbers are well recognized to increase the risk of overfitting and instability of apparent model performance estimates; this supports the use of parsimonious prespecified models and cautious interpretation of incremental predictive gains. Third, evaluation of prediction models should not rely on discrimination alone; broader assessment of model performance and validation is recommended when considering the contribution of an additional predictor (29). Thus, ROTEM may still be clinically informative by identifying biologically distinct hemostatic phenotypes relevant to management, even if it does not materially improve mortality discrimination in this dataset.

Accordingly, the clinical role of ROTEM in neonatal sepsis may be better framed as an adjunctive whole-blood hemostatic assessment that may help characterize coagulation patterns and support hemostatic evaluation in selected high-risk patients, rather than as a universal mortality predictor for all septic neonates (29, 34). This is consistent with neonatal literature emphasizing that viscoelastic results in this age group require population-appropriate interpretation, as neonatal hemostasis differs from that of older children and adults and reference ranges remain limited (17). It is also consistent with review data indicating that sepsis-associated coagulopathy evolves dynamically, that ROTEM/TEG findings vary with disease stage and timing of testing, and that sequential measurement—especially when combined with conventional coagulation assays—may better depict progression from early coagulation activation to later hypocoagulability/DIC than a single early test alone (24). Future studies should therefore examine whether serial viscoelastic assessment, integrated with conventional coagulation testing, improves characterization of disease evolution and better informs hemostatic management in neonatal sepsis (24, 34). This interpretation is also supported by recent neonatal data suggesting that coagulation abnormalities and viscoelastic alterations are closely linked to disease severity and organ dysfunction rather than functioning as standalone mortality predictors (26, 36). Therefore, ROTEM may be most valuable when integrated with clinical severity assessment tools such as nSOFA rather than used in isolation. The modest incremental value of FIBTEM MCF observed in our study should therefore not be interpreted as evidence against the clinical utility of ROTEM. Rather, it suggests that when a strong organ dysfunction score such as nSOFA is already incorporated into the baseline model, the additional discriminatory gain obtained from a single viscoelastic parameter may be limited. ROTEM may remain valuable for characterizing hemostatic phenotype, identifying clinically relevant coagulation abnormalities, and supporting individualized hemostatic assessment even when improvements in mortality discrimination are modest.

The high proportion of culture-negative sepsis in this cohort (75.6% of cases) is consistent with the clinically defined nature of neonatal sepsis cohorts and with literature indicating that microbiological cultures may be less conclusive in early sepsis care, particularly when specimens are obtained while patients are already receiving antimicrobial therapy; in addition, culture results often require 2–3 days to finalize (37). Culture status did not differ by survival in our cohort, suggesting that short-term mortality may be driven more by the magnitude of host response and organ dysfunction than by microbiologic confirmation alone. This is compatible with pediatric sepsis frameworks, which define sepsis-associated organ dysfunction on the basis of severe infection with cardiovascular and/or noncardiovascular organ dysfunction and explicitly include cases with suspected as well as confirmed invasive infection (20). Accordingly, the predominance of culture-negative cases in our study should be interpreted not simply as a microbiologic limitation, but also as a feature of a clinically defined sepsis cohort, which may encompass heterogeneous infectious etiologies and thereby broaden the observed variability in coagulation phenotypes.

The recently proposed Phoenix criteria for pediatric sepsis and septic shock provide an important contemporary framework for identifying infection-associated organ dysfunction in children and incorporate coagulation dysfunction among their organ-system domains (38). Although term neonates were represented during the derivation and validation of the Phoenix criteria, the framework has not been specifically developed or validated for hospitalized neonatal intensive care unit (NICU) populations, particularly newborns who remain hospitalized after birth, whereas neonatal-specific tools such as the neonatal Sequential Organ Failure Assessment (nSOFA) were specifically designed for neonatal critical care. Accordingly, the present study adopted the EMA 2010 neonatal sepsis criteria together with the prespecified nSOFA score because these neonatal-specific approaches are more directly aligned with our study population, clinical setting, and predefined study protocol, rather than because of neonatal age alone. Moreover, our objective was not to compare sepsis classification frameworks but rather to characterize coagulation abnormalities and ROTEM-derived viscoelastic profiles and to evaluate their prognostic relevance beyond established measures of neonatal illness severity. Future studies should evaluate the performance of Phoenix-based approaches in hospitalized neonatal sepsis cohorts and determine whether they provide complementary prognostic information alongside neonatal-specific severity assessment tools such as nSOFA.

Several limitations should be considered. First, this was a single-center study conducted in a tertiary referral neonatal unit and included only term neonates, which may limit generalizability to preterm populations and other healthcare settings. Second, coagulation tests and ROTEM analyses were performed within 24 h of sepsis diagnosis; however, the exact timing relative to blood culture acquisition, antibiotic administration, and early resuscitation measures was not prospectively recorded. Consequently, temporal effects of early therapeutic interventions on coagulation parameters could not be fully assessed. Third, missing data, particularly for ROTEM variables, were influenced by operational constraints and may have introduced selection bias. Fourth, the relatively small number of deaths limited statistical power, constrained multivariable modeling, and increased the possibility of model instability and overfitting despite the use of prespecified parsimonious models. Fifth, model performance was primarily evaluated using ROC-based discrimination. Additional measures such as precision-recall analysis, including the area under the precision-recall curve, were not assessed and may provide complementary information in datasets with class imbalance. Furthermore, internal validation procedures such as bootstrap resampling or cross-validation were not performed; therefore, model performance estimates should be interpreted cautiously until confirmed in independent cohorts. Sixth, interventions including transfusion practices and supportive therapies were not standardized, and residual confounding cannot be excluded. Seventh, although both early-onset sepsis and late-onset sepsis were included, the study was not designed or powered to perform detailed stratified analyses according to sepsis onset. Consequently, potential differences in microbiological epidemiology, including the distribution of Gram-positive and Gram-negative pathogens among culture-positive cases, as well as differences in coagulation abnormalities and ROTEM profiles between early-onset sepsis and late-onset sepsis, could not be adequately assessed. Future multicenter studies with larger numbers of microbiologically confirmed infections should evaluate these subgroup-specific characteristics in greater detail. Finally, only short-term mortality was evaluated; longer-term outcomes, including neurodevelopmental impairment and functional status, were not available.

## Conclusions

Among term neonates with sepsis, mortality was associated with early organ dysfunction severity and was accompanied by a consistent hypocoagulable pattern across conventional coagulation tests and ROTEM—most prominently reduced clot propagation and clot strength, including fibrin-based clot firmness. Early organ dysfunction severity, as captured by nSOFA, was the dominant determinant of short-term mortality. While nSOFA provided strong baseline prognostic discrimination, FIBTEM MCF showed a potentially clinically meaningful association with mortality but only modest and statistically non-significant incremental discrimination beyond nSOFA and INR. These findings suggest that viscoelastic testing may provide complementary pathophysiologic information, but its added prognostic value appears limited in this setting. Further multicenter studies incorporating serial viscoelastic assessment are needed to clarify its clinical utility in risk stratification and hemostatic management.

## Data Availability

The raw data supporting the conclusions of this article will be made available by the authors, without undue reservation.
